# Influence of Ni Contents on Microstructure and Mechanical Performance of AlSi10Mg Alloy by Selective Laser Melting

**DOI:** 10.3390/ma16134679

**Published:** 2023-06-28

**Authors:** Hui Wang, Like He, Qingyong Zhang, Yiqing Yuan

**Affiliations:** 1 Fujian Key Laboratory of Intelligent Machining Technology and Equipment, Fujian University of Technology, Fuzhou 350118, China; 2200101001@smail.fjut.edu.cn (L.H.); zhqy@fjut.edu.cn (Q.Z.); yqyuan@tongji.edu.cn (Y.Y.); 2 School of Mechanical and Automotive Engineering, Fujian University of Technology, Fuzhou 350118, China

**Keywords:** selective laser melting, Ni/AlSi10Mg, Si network, Al_3_Ni nanoparticles, mechanical properties

## Abstract

To improve the tensile strength and wear resistance of AlSi10Mg alloys, a novel in situ synthesis method of selective laser melting (SLM) was used to fabricate the Ni-reinforced AlSi10Mg samples. The eutectic Si networks formed around the *α*-Al crystals by diffusion and transportation via Marangoni convection in the SLM process. Moreover, the XRD and TEM results verified that the Al_3_Ni nanoparticles were created by the in situ reaction of the Ni and aluminum matrix in the Ni/AlSi10Mg samples. Therefore, the microstructure of the Ni-containing alloys was constituted by the *α*-Al + Si network + Al_3_Ni phases. The dislocations accumulated at the continuous Si network boundaries and cannot transmit across the dislocation walls inside the Si network. SEM results revealed that the continuity and size of eutectic Si networks can be tailored by adjusting the Ni contents. Furthermore, the Al matrix also benefited from the Al_3_Ni nanoparticles against the dislocation movement due to their excellent interfacial bonding. The 3Ni-AlSi10Mg sample exhibited high mechanical properties due to the continuous Si networks and Al_3_Ni nanoparticles. The tensile strength, elongation, Vickers hardness, friction coefficient, and wear volumes of the 3Ni-AlSi10Mg samples were 401.15 ± 7.97 MPa, 6.23 ± 0.252%, 144.06 ± 0.81 HV, 0.608, 0.11 mm^3^, respectively, which outperformed the pure AlSi10Mg samples (372.05 ± 1.64 MPa, 5.84 ± 0.269%, 123.22 ± 1.18 HV, 0.66, and 0.135 mm^3^).

## 1. Introduction

Aluminum alloys have been widely used in aerospace, the automotive industry, and biomedicine due to their excellent forming ability [1]. Instruments made of aluminum alloys have a low weight, a low thermal expansion coefficient, and excellent corrosion resistance. However, aluminum alloys show low tensile strength and poor wear resistance, which greatly hinders their applications in multiple fields [2].

Selective laser melting (SLM), also known as laser-based powder bed fusion (LPBF), is an additive manufacturing method. Compared with conventional manufacturing methods, the SLM process allows for free-form manufacturing and has the potential to enable time and cost savings for complex and customized parts. The increasing demand for producing AlSi10Mg alloys with complex shapes promoted the research to fabricate the AlSi10Mg alloys via the SLM process. Moreover, the SLM process is beneficial to improve the mechanical properties of the AlSi10Mg alloys due to the formation of a non-equilibrium supersaturated solid solution at an extremely fast cooling rate (∼10^3^–10^7^ K/s) [3]. Previous works in the literature showed that the peak stresses and average activation energy of the additively manufactured material were lower than their hot-forging counterparts [4]. Furthermore, the input parameters of the SLM process should be properly chosen to ensure the mechanical and microstructure properties of the final produced component [5]. The above research highlighted the capability of additive manufacturing as a cost-effective and energy-saving production method for the Ni/AlSi10Mg.

AlSi10Mg is a typical heat-treatable gravity cast (GC) alloy and is widely used due to its excellent casting and welding performance. However, the strength, elongation, and wear properties of the AlSi10Mg alloys are insufficient. Aiming to improve the mechanical performance of the AlSi10Mg alloys, scholars have used additions, including LaB_6_ and BN, as grain-refining agents. Tan et al. [6] investigated the effect of the LaB_6_ nanoparticles on the microstructural evolution and mechanical performance of the SLMed AlSi10Mg samples. The selective laser melting (SLM) process was used to obtain the freeform AlSi10Mg samples coupled with high mechanical properties. The grains of the AlSi10Mg were refined due to the heterogeneous nucleation of Al on the LaB_6_ nanoparticles during solidification. Though the elongation of the AlSi10Mg samples was improved significantly, the addition of the LaB_6_ nanoparticles had a very marginal effect on the tensile strength of the AlSi10Mg alloys at all addition levels, from 0.05 wt.% to 2 wt.%. Another approach proposed to improve the mechanical properties of the AlSi10Mg alloy is the use of reinforcing particles such as TiC, TiN, etc. Gao et al. [7] fabricated TiN/AlSi10Mg nanocomposites by using the SLM process. The hardness of the samples increased from 126 HV_0.1_ to 145 HV_0.1_ with the TiN contents of 2 wt.%. However, the interfacial bonding between the Al matrix and reinforcements is usually weak, and the weak point will degrade the mechanical performance of the samples. The in situ alloying process is another effective method to improve the mechanical properties of the AlSi10Mg alloy. However, to the best of our knowledge, far too little attention has been paid to the development of new AlSi10Mg alloys through the in situ alloying process. Since the trace element Ni can react with the Al matrix to form the precipitation phases, this work aimed to assess the feasibility of producing a Ni-modified AlSi10Mg alloy through the SLM method.

Previous studies have focused mainly on the wettability and distribution of the reinforcement particles, as well as the heat treatment of the AlSi10Mg alloys, while less attention has been paid to the influence of the inclusion contents on the structure of the AlSi10Mg samples. In this study, Ni/AlSi10Mg samples were fabricated via SLM with 0 to 5 wt.% Ni additions. The literature suggested that Al-based composites reinforced by particulate metallic materials could be a promising research direction in additive manufacturing, as the metal particles enhanced light absorption [8]. Thus, research on the fabrication of the Ni/AlSi10Mg alloys via SLM is necessary. To investigate the effect of the addition of Ni nanoparticles on the microstructure and mechanical properties, the microstructure, phase identification, and nanoparticle’s state of the SLMed samples were analyzed by SEM, XRD, and TEM, respectively. The ultimate tensile strength, elongation, Vickers hardness, coefficient of friction, and wear volumes were also measured.

## 2. Experimental Procedures

### 2.1. Materials Preparation

**Figure 1:**
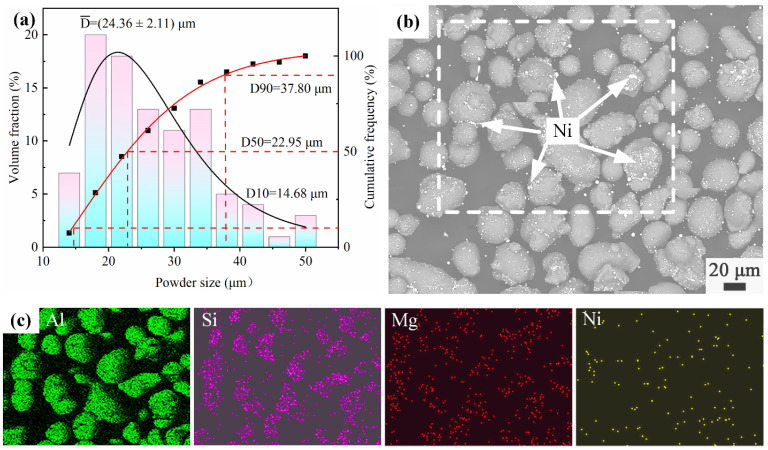
(**a**) Size-distribution histogram of AlSi10Mg powders, (**b**) SEM morphology of 3Ni-AlSi10Mg composite powders, and (**c**) EDS elements distribution of the white wireframe area in (**a**).

Gas-atomized AlSi10Mg powders (provided by Sichuan Air Sky Additive Manufacturing Co., Ltd., Chengdu, China) and Ni nanoparticles with high purity (99.9%, provided by Shanghai Rhawn Reagent Co., Ltd., Shanghai, China) were used as the raw materials. The size-distribution histogram of the AlSi10Mg powders is presented in Figure 1a. The diameter of the AlSi10Mg powders ranged from 13 to 53 μm, and the average diameter was 24.36 ± 2.11 μm. The diameter of the Ni nanoparticles ranged from 20 to 100 nm. The addition of the Ni nanoparticles to AlSi10Mg was set to be 0, 1, 3, and 5 wt.%, sequentially. The composites were then mixed uniformly in the planetary ball mill (Miqi Instrument Equipment Co., Ltd., Changsha, China) under the protection of argon. The material-to-ball-mass ratio was 10:1, the rotation speed was 300 r/min, and the milling time was 3 h. To avoid the additional chemical reaction, the ball-milling process was paused for 10 min every 1 h to cool down the system. The mixed powders were then analyzed using the Nova Nano field-emission SEM 450 and energy-dispersive X-ray spectroscopy (EDS). The morphology of the 3Ni-AlSi10Mg composite powders is shown in Figure 1b. It is shown that the Ni nanoparticles uniformly adhered to the surface of AlSi10Mg powders, and all the composite powders remained nearly spherical after ball milling. The elements distribution analysis of the as-mixed 3Ni-AlSi10Mg powders is shown in Figure 1c; it can be further confirmed that the Ni nanoparticles were distributed uniformly within the AlSi10Mg powder.

### 2.2. Sample Production

**Figure 2:**
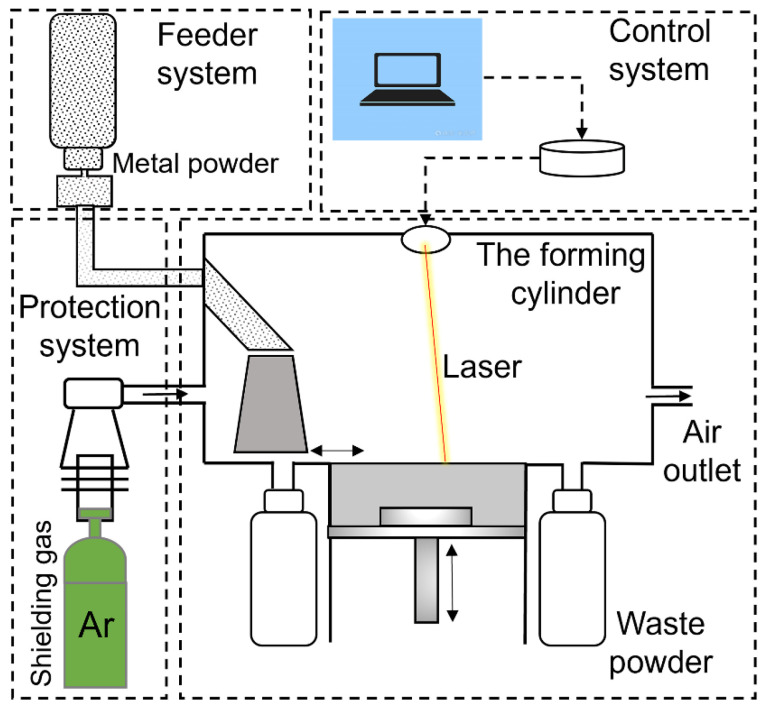
Schematic diagram of the selective laser melting (SLM) system.

**Figure 3:**
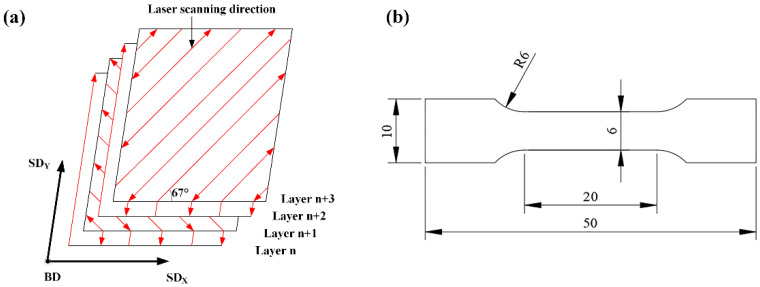
(**a**) Scanning strategy of the SLM process and (**b**) size of the Ni/AlSi10Mg samples for the tensile test (unit in mm).

The SLM125HL system (SLM Solutions, Lubeck, Germany) with a building envelope of 125 × 125 × 125 mm^3^ was used to fabricate the Ni/AlSi10Mg composite samples. The system was equipped with an IPG fiber laser with a wavelength of 1070 nm, a maximum power of 400 W, and a spot diameter of 70 μm. The schematic diagram of the SLM system is shown in Figure 2. In this study, the printing parameters were selected as follows: a laser power of 350 W, scanning speed of 1850 mm/s, hatch spacing of 130 μm, and layer thickness of 30 μm, as shown in Table 1. The authors took the standard process package of pure AlSi10Mg processed by SLM 125HL (laser power of 350 W, scanning speed of 1650 mm/s, hatch spacing of 130 μm, and layer thickness of 30 μm) and the literature as a reference [9,10,11,12]. A multidirectional meander scan strategy was used in which the rotation angle between neighboring layers was 67° to reduce the residual stress and improve the crack-growth resistance, as shown in Figure 3a. Cubic samples with the dimensions 8 mm × 8 mm × 8 mm were prepared for the Vickers-hardness and wear-resistance tests. Rectangular-shaped samples with the dimension 50 mm length × 10 mm width × 2.5 mm thickness were prepared for the tensile test. The final shape was cut by wire EDM according to ASTM E8M standard [13], as shown in Figure 3b.

**Figure 4:**
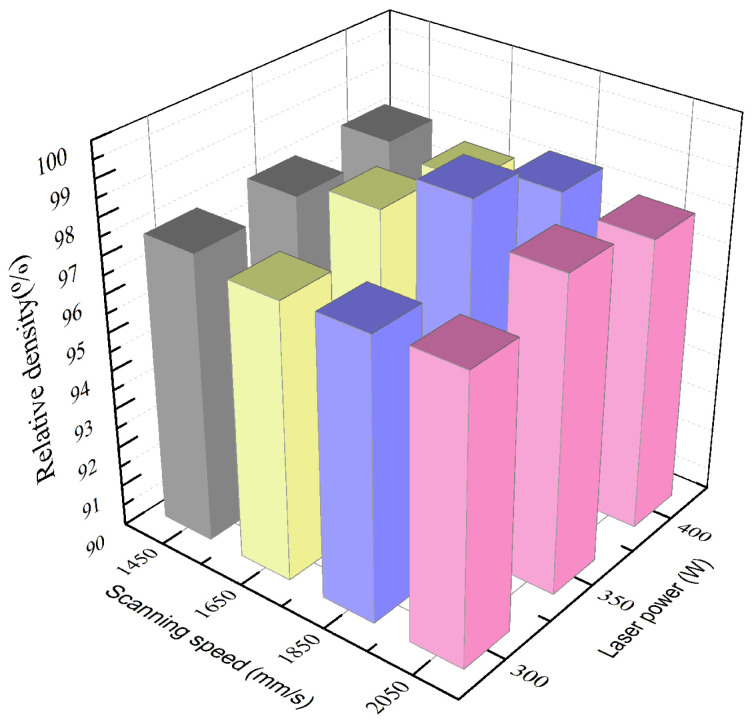
The relative densities of Ni/AlSi10Mg samples prepared at different laser powers and scanning speeds.

In our preliminary experiments, detailed research on the effect of different printing parameters was conducted. The relative densities of Ni/AlSi10Mg samples prepared at different laser powers and scanning speeds are shown in Figure 4. It can be seen that the AlSi10Mg samples treated at the laser power of 350 W and scanning speed of 1850 mm/s showed the highest relative densities among all the samples. Therefore, we chose an optimal laser power of 350 W and a scanning speed of 1850 mm/s to conduct more in-depth research. That is, this paper further studied the effect of different Ni contents on the microstructure and mechanical properties of Ni/AlSi10Mg samples at an optimal laser power of 350 W and scanning speed of 1850 mm/s.

### 2.3. Relative Density Test

The Archimedes drainage method was used to determine the density of the Ni/AlSi10Mg samples. The density (*ρ*) of the samples was calculated by *ρ* = *M*_s_/*m⸱ρ*_w_, where *M*_s_ is the weight of the sample in the air, *m* is the weight difference of the container before and after putting the sample in the water, and *ρ*_w_ is the density of the distilled water. The theoretical density (*ρ*_α_) of the Ni/AlSi10Mg composite sample was calculated using the linear mixing rule based on the weight percentage of the Ni and AlSi10Mg. A theoretical density of 8.9 g/cm^3^ for Ni and a theoretical density of 2.68 g/cm^3^ for AlSi10Mg were used. The relative density (*D*) of the samples was calculated by *D* = *ρ*/*ρ*_α_⸱100%. The average relative densities were calculated from five samples after removing the maximum and the minimum values.

### 2.4. Characterization and Mechanical Evaluation

Room-temperature tensile tests were conducted using an INSTRON 2382 tester (Boston, MA, USA) at a displacement velocity of 0.6 mm/min. Friction and wear tests were conducted on the Bruker UMT-TriboLab device (Billerica, MA, USA). A Si_3_N_4_ ball with a diameter of 10 mm was chosen as the wear couple. The Si_3_N_4_ ball rubbed back and forth at a reciprocating frequency of 2 Hz in a stroke of 4 mm. The experiment was performed at a normal load of 3 N and a wear time of 15 min. Each sample was sanded with sandpaper of different grit sizes and polished before testing. The coefficient of friction (COF) was automatically recorded by the tribometer. Finally, the wear volume and the change in surface profile after wear tests were measured and analyzed using a white-light interference microscope. The Vickers hardness of the samples was measured using a digital Vickers hardness tester (THV-10D, Teshitest Co., Ltd., Shanghai, China), using a load of 1000 g and a dwell time of 10 s. Seven points were tested for each sample, and the average value after removing the maximum and the minimum value was adopted.

An optical microscope (KH1300, Tokyo, Japan) was used to characterize the metallographic images of the samples. The samples were polished and etched with Keller’s reagent (1% HF, 1.5% HCl, 2.5% HNO_3_, and 95% H_2_O by volume) for 15 s before testing. Then, the field emission scanning electron microscope (Nova NanoSEM 450, FEI, Hillsboro, OR, USA) was used to analyze the microstructure morphology of the samples. The regions of interest were analyzed by an energy-dispersive X-ray spectrometer (EDS). The size of nanoparticles and the crystallinity were further characterized by transmission electron microscopy (TEM, JEM-2100, JEOL Ltd., Tokyo, Japan). The phase identification of the samples was analyzed by the X-ray diffractometer (XRD, with Cu Kα radiation (λ = 1.5418 Å), D8 Advance, Bruker Inc., Stuttgart, Germany) between 10° and 90°, at a step width of 0.02° and scan speed of 6°/min. The working voltage and current used were 40 kV and 40 mA, respectively.

## 3. Results and Discussion

### 3.1. Densification Behavior and Phase Recognition

**Figure 5:**
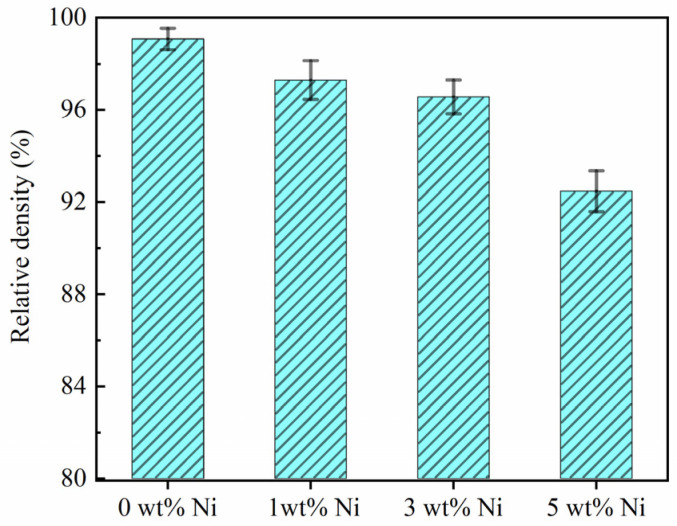
Relative density of Ni/AlSi10Mg samples with different Ni contents.

Figure 5 shows the relative density of Ni/AlSi10Mg samples with different Ni contents. The relative density of pure AlSi10Mg samples fabricated via SLM reached 99.07 ± 0.46%. The slight porosity should be attributed to the metallurgical pores where gases were trapped within the melt pool or evolved from the powder during consolidation [14]. Thereafter, the relative density of the SLM-processed Ni/AlSi10Mg samples decreased when increasing the Ni content. The relative densities corresponding to the Ni content of 1 wt.%, 3 wt.%, and 5 wt.% were 97.29 ± 0.84%, 96.56 ± 0.74%, and 92.47 ± 0.88%, respectively.

**Figure 6:**
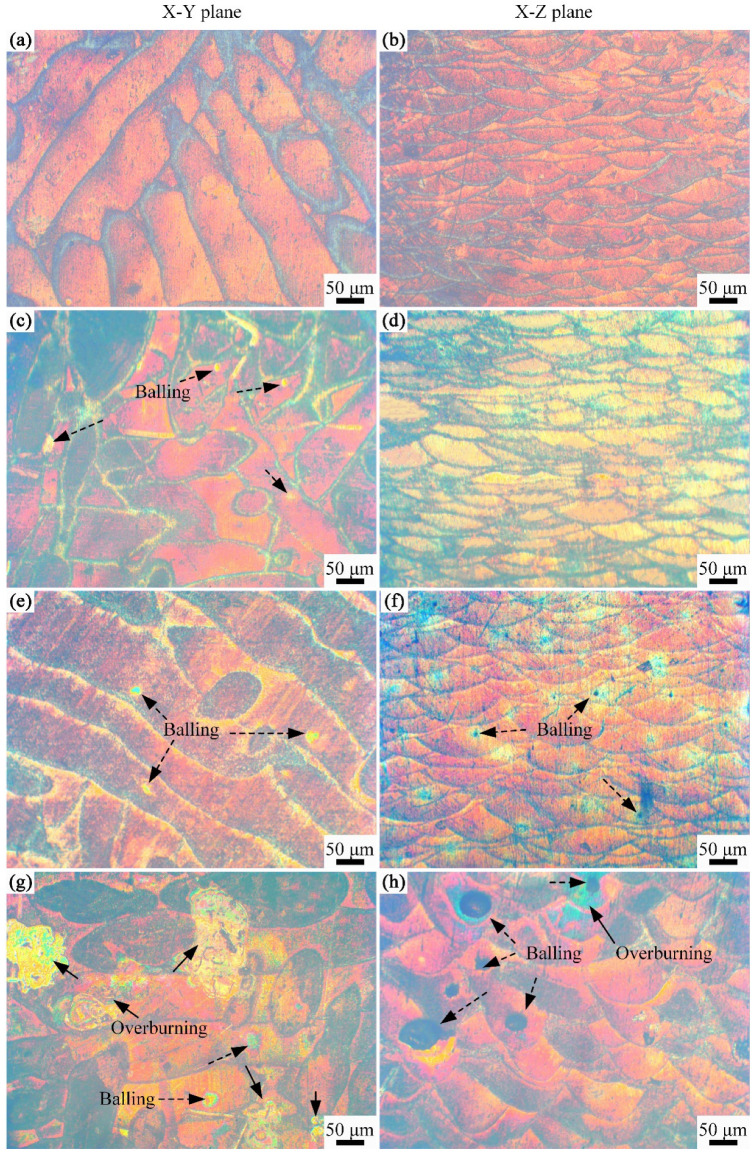
OM images of horizontal X-Y plane and vertical X-Z plane of Ni/AlSi10Mg samples with different Ni contents: (**a**,**b**) 0 wt.%, (**c**,**d**) 1 wt.%, (**e**,**f**) 3 wt.%, and (**g**,**h**) 5 wt.%.

**Figure 7:**
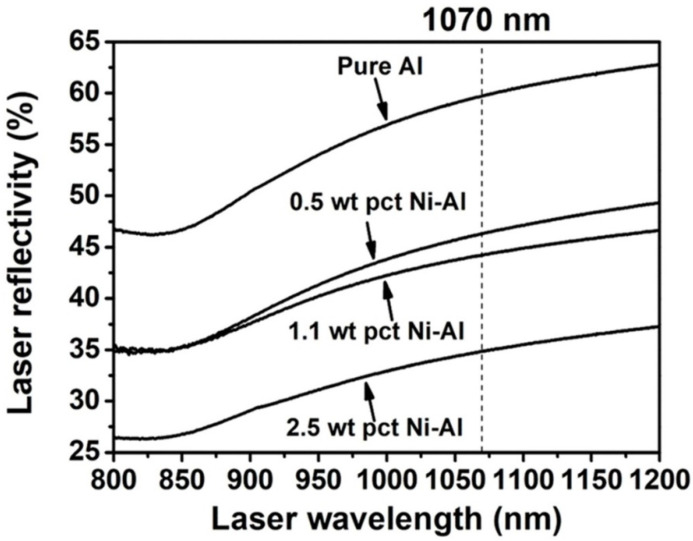
The laser reflectivity of pure Al powder and Al powder with different Ni contents (Reproduced from [15]).

Figure 6 shows the OM images of the horizontal X-Y plane and vertical X-Z plane of Ni/AlSi10Mg samples with different Ni contents. The laser scanning track was observed at the X-Y plane of the samples, and the molten pools with fish-scale patterns were observed at the X-Z plane of the samples. The pure AlSi10Mg samples revealed a surface with metallic luster and clear tracks, as shown in Figure 6a. However, balling and over-burning were found on both the X-Y and X-Z planes of the samples when the Ni content increased in the Ni/AlSi10Mg composites. Figure 7 shows the effect of the Ni content on the laser reflectivity of Al powders. Geng et al. [15] verified that the laser absorptivity of Al powders would be affected by the Ni contents significantly. At the laser wavelength of 1070 nm used in this study, the laser absorptivity of 2.5 wt.% Ni-added Al powders was ∼65%, which was much higher than that of the pure Al powders (∼40%). Because of the increase in the laser absorptivity, the powders absorbed more laser energy and achieved higher temperatures in the molten pool. Then, the enhanced convection of the molten pool would cause droplets to splash, which then solidified into metal balls [16,17], as indicated by the arrows in Figure 6c,e. When the next layer was built, the gaps among the balling were difficult to fill with the melted alloys and were consequently kept as pores in the samples [18]. The excessive energy also caused serious over-burning of the surfaces in 5Ni-AlSi10Mg samples, as indicated by the arrows in Figure 6g. Then, the balling and over-burning tended to affect the pavement quality of the next powder layer; therefore, the density of the 1Ni-, 3Ni-, and 5Ni-AlSi10Mg samples decreased as the pavement quality became worse layer by layer. Similar results were verified by the studies of Chen et al. and Kuai et al. [11,19]. Thus, the lower relative density of the Ni/AlSi10Mg samples can be attributed to the balling and over-burning of the surface caused by the increase of the laser absorptivity by Ni nanoparticles.

As shown in Figure 6b,d,f,h, the surfaces along the building direction (vertical X-Z plane) clearly exhibited fish-scale structures, which were melt-pool boundaries. Each scale was a semi-cylindrically shaped element after the melting of several powder particles via Gaussian laser scanning. No holes or other defects were found in the pure AlSi10Mg samples, as shown in Figure 6b. Similarly, balling and over-burning could be observed in the lateral surfaces of the Ni/AlSi10Mg samples. In addition, as presented in Figure 6d,f,h, the fish scales became slightly wider and deeper for the Ni/AlSi10Mg samples, and this result should be attributed to the higher energy density induced in the material.

**Figure 8:**
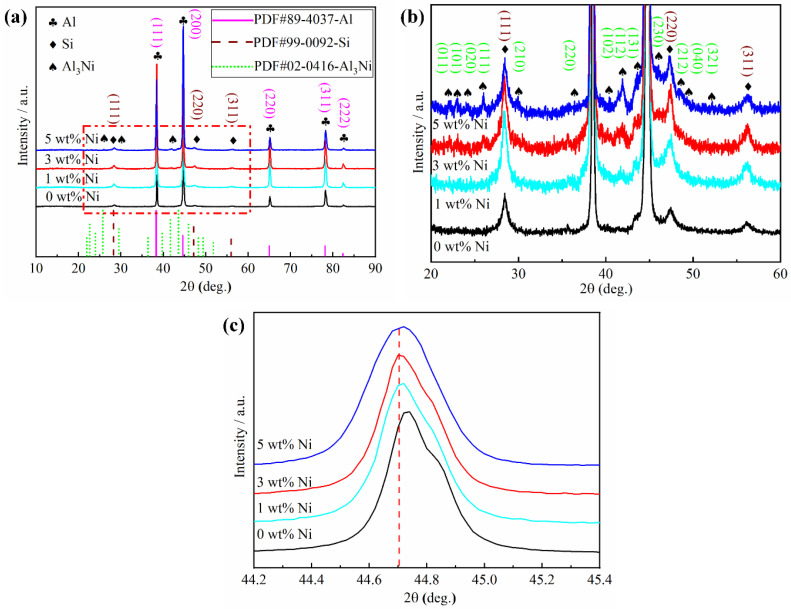
(**a**) X-ray diffraction patterns of Ni/AlSi10Mg samples with different Ni contents; magnified area of Ni/AlSi10Mg samples (**b**) in the dotted box of (**a**,**c**) in the range of 2*θ* = 44.2°∼45.4°.

Figure 8 shows the XRD pattern of Ni/AlSi10Mg samples with different Ni contents. The diffraction peaks of the *α*-Al phase can be easily observed in all the samples, as shown in Figure 8a. The preferred orientation of *α*-Al in AlSi10Mg was (111) and (200), and the orientation of *α*-Al in the (200) direction was the strongest. During the SLM process, a part of Si was precipitated in the Al matrix due to the eutectic reaction of L→*α*-Al + Si. The remaining Si dissolved in the Al matrix, leading to the weak intensity of the Si peaks, as shown in Figure 8b. It can be seen that the peaks of Si were comparatively higher in the Ni/AlSi10Mg samples than that in the pure AlSi10Mg samples. This indicates significant precipitation of the Si particles in the Ni/AlSi10Mg samples. No clear peaks of Mg were found in the XRD pattern because the content of Mg in AlSi10Mg was very low (<0.45 wt.%). Based on the close-up view between 20° and 60° shown in Figure 8b, the intermetallic Al_3_Ni was formed in the Ni-added AlSi10Mg samples. The absence of Ni peaks suggests that all Ni powders reacted with Al during the SLM process to form the Al_3_Ni nanoparticles. When the Ni increased from 1 wt.% to 5 wt.%, the diffraction peak angle of *α*-Al (200) shifted to a small value to the left, as shown in Figure 8c. This is because the *α*-Al matrix existed in the form of a replacement solid solution, and the atomic radius of Ni (0.149 nm) was much larger than that of Al (0.118 nm). When the Al atom was substituted by the Ni atom, lattice distortion was generated, and the lattice parameter of the *α*-Al matrix became larger, leading to the left shifting of the peak angle of α-Al (200). However, the solubility of Ni in solid Al was as low as 0.04 wt.% [20,21]; thus, the peak angles of α-Al (200) in the 1Ni-, 3Ni-, and 5Ni-AlSi10Mg samples did not differ too much from each other.

### 3.2. Microstructure Characterization

**Figure 9:**
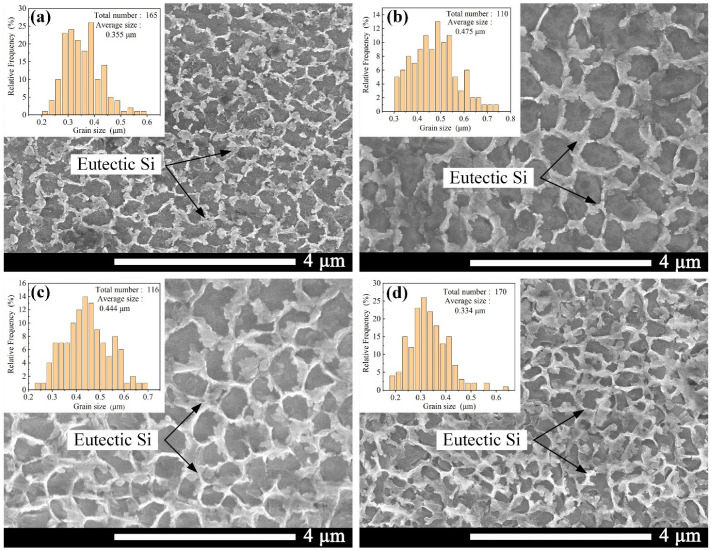
SEM images of the surface perpendicular to the building direction (X-Y plane) of Ni/AlSi10Mg samples with different Ni contents: (**a**) 0 wt.%, (**b**) 1 wt.%, (**c**) 3 wt.%, and (**d**) 5 wt.%. Insets show the size-distribution histograms of the Si networks.

Figure 9 shows the SEM images of the top surface (X-Y plane) of Ni/AlSi10Mg samples with different Ni contents. It can be observed that all of the samples showed cellular organization, in which the dark gray primary *α*-Al matrix was decorated with a white fiber eutectic Si network. The primary *α*-Al grew here in the form of cell crystals. In the SLM process, the large temperature gradient induced a high degree of undercooling and a high gradient of surface tension, hence leading to Marangoni convection. The eutectic Si element was evenly distributed around the *α*-Al crystals by diffusion and transportation via Marangoni convection, which gave rise to the formation of the Si network [22,23].

**Figure 10:**
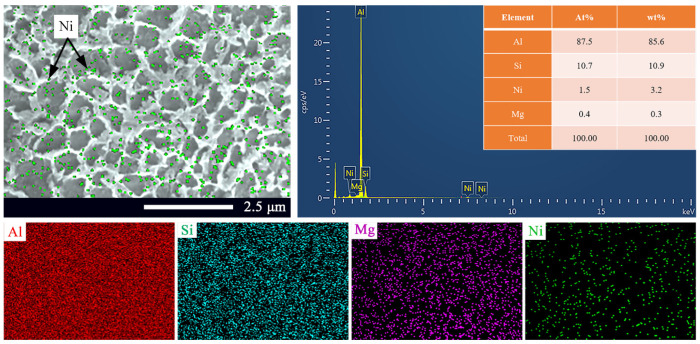
EDS analysis of the surface perpendicular to the building direction (X-Y plane) of the 3Ni-AlSi10Mg sample.

On the other hand, the morphologies of the Si networks differed from each other in the SLM Ni/AlSi10Mg samples. The diameters of the Si networks were measured using the Image-Pro Plus image analysis software. The corresponding size-distribution histograms are shown in the insets in Figure 9a–d. The average sizes of the Si networks were 0.355, 0.475, 0.444, and 0.334 μm, respectively, for the pure AlSi10Mg, 1Ni-AlSi10Mg, 3Ni-AlSi10Mg, and 5Ni-AlSi10Mg SLMed samples. As shown in Figure 9a,b, it is worth noting that the average size of the Si networks of the 1Ni-AlSi10Mg sample was much larger than that of the pure AlSi10Mg samples. With the addition of the Ni content, the laser energy input into the molten pool was relatively denser, which allowed the liquid phase to exist longer, leading to the formation of coarser Si networks. As the Ni content further increased from 1 wt.% to 5 wt.%, the growth of the Si networks was inhibited, and the average size decreased from 0.475 μm to 0.334 μm. According to the studies by Gao et al. and Wei et al. [24,25,26], the introduced nanoparticles can produce heterogeneous nucleation sites, thereby inhibiting the growth of *α*-Al cells and favoring the cell refinement. Since the addition of Ni nanoparticles can promote the heterogeneous nucleation of *α*-Al, *α*-Al in the liquid phase preferentially nucleated and grew up. Moreover, the Ni particles were mostly located along the cell boundaries, as shown in Figure 10, and the Si networks were then refined due to the limitation of cellular *α*-Al growth due to the Ni pinning at the cell boundary and the introduction of nucleation sites.

It is also suggested that the addition of Ni would increase the migration of the Si from the supersaturated Al matrix toward the pre-existing Si network and eventually promote the formation of denser Si networks, as shown in Figure 9b–d. This result was also related to the enhanced peak intensity of Si in the 1 Ni-, 3Ni-, and 5Ni-AlSi10Mg samples in the XRD pattern in Figure 8.

Figure 10 shows the EDS analysis of the X-Y plane of the 3Ni-AlSi10Mg sample. The element distribution was uniform due to the extremely fast cooling rate, which was as fast as 10^3^ K/s. Except for the eutectic Si network, some Si particles also precipitated out in the *α*-Al crystals due to their limited solid solubility. SEM images coupled with EDS results of 3Ni- AlSi10Mg showed that the Ni elements were distributed partly inside the *α*-Al cells, while most of them were found to be located along the *α*-Al cells’ boundaries.

**Figure 11:**
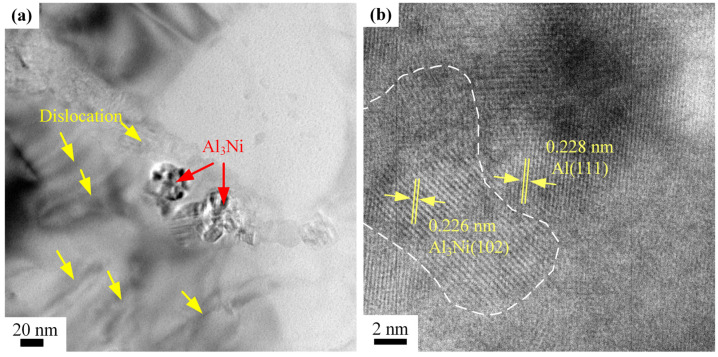
TEM and HRTEM images of the 3Ni-AlSi10Mg sample: (**a**) Al_3_Ni nanoparticles and (**b**) HRTEM images of the interface between the Al matrix and the Al_3_Ni nanoparticle.

Figure 11a is a typical TEM image clearly showing the existence of nanosized Al_3_Ni particles (indicated by red arrows) inside the *α*-Al matrix. The results indicated that the high temperature of the laser irradiation could melt the Ni particles and cause a reaction between the molten Ni and aluminum matrix. Consequently, Ni was transformed into Al_3_Ni intermetallic chunks. The size of the Al_3_Ni phase was roughly 30 nm, which was close to the size of the Ni nanoparticles. The XRD peaks of the Al_3_Ni phase were enhanced in the 5Ni-AlSi10Mg sample due to its higher Ni contents, as shown in Figure 8b. A large number of dislocations were observed around the Al_3_Ni nanoparticles by the yellow arrows, as shown in Figure 11a.

The bonding interface between the Al matrix and Al_3_Ni particles was further determined by HRTEM, as shown in Figure 11b. The lattice spacings of 0.228 nm (corresponded to Al (111)) and 0.226 nm (corresponded to Al_3_Ni (102)) could be identified clearly. The interface between the Al_3_Ni nanoparticle and the Al matrix was well-bound due to the in situ reaction bonding. According to the Bramfitt lattice-matching theory, the unmatched ε < 5% is a coherent interface, 5% < ε < 15% is a semi-coherent interface, and ε > 15% is an incoherent interface [27]. The lattice mismatch (ε) between (102)_Al3Ni_ and (111)_Al_ is 0.9%, which indicates that (102)_Al3Ni_ and (111)_Al_ are coherent interfaces. Good interfacial bonding between Al_3_Ni nanoparticles and the Al matrix is a key factor to ensure load-transfer efficiency [28].

### 3.3. Mechanical Property

**Figure 12:**
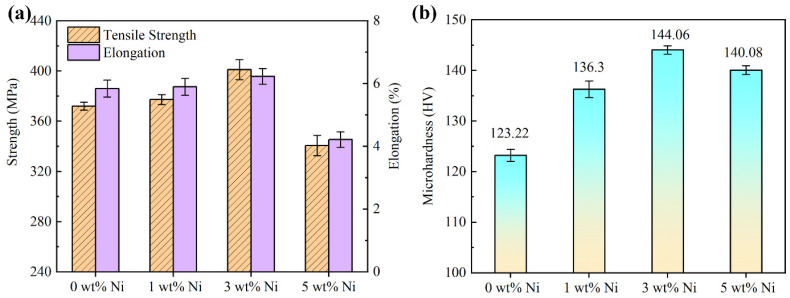
Mechanical properties of Ni/AlSi10Mg samples with different Ni contents: (**a**) tensile strength and elongation and (**b**) microhardness.

**Figure 13:**
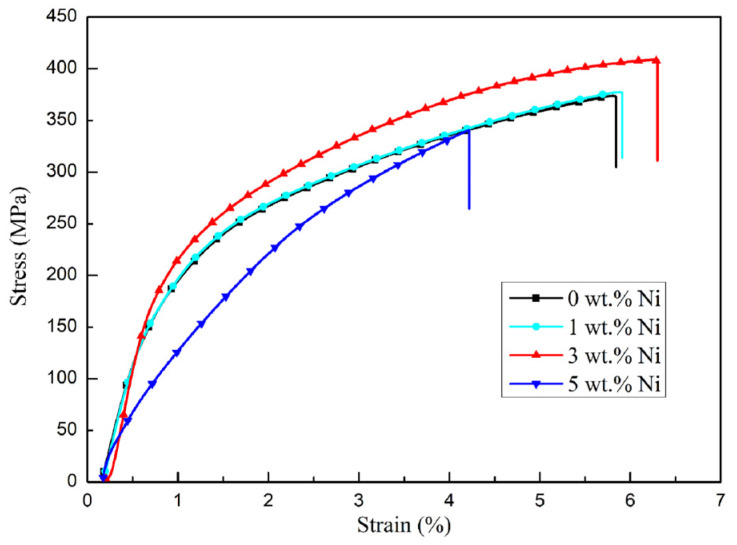
The stress–strain diagram of the Ni/AlSi10Mg samples with different Ni contents.

Figure 12 shows the tensile strength, elongation, and Vickers hardness of Ni/AlSi10Mg samples with different Ni contents. The detailed mechanical properties are listed in Table 2, and the stress–strain diagram of the Ni/AlSi10Mg samples with different Ni contents is shown in Figure 13. The mechanical properties of the Ni/AlSi10Mg samples show a trend of first increasing and then decreasing with the increasing Ni contents, as shown in Figure 12 and Figure 13. When the Ni content was 3 wt.%, the tensile strength, elongation, and Vickers hardness of the samples achieved the peak values of 401.15 ± 7.97 MPa, 6.23 ± 0.252%, and 144.06 ± 0.81 HV, respectively.

The high tensile strength of the 1Ni- and 3Ni-AlSi10Mg samples should be due to their fine Si networks, as shown in Figure 9c. Chen et al. [29] and Zhao et al. [30] demonstrated the effectiveness of the Si network in hindering dislocation movement and identified the Orowan looping as the dominant strengthening mechanism for AlSi10Mg alloys. During the deformation process, the dislocation could be pinned by the nanosized Si particles at the Si network boundaries, which activated the Orowan mechanism and produce a strong strain-hardening effect [31]. The refined Si network in the 3Ni-AlSi10Mg sample reduced the distance between Si particles, which can reduce the dislocation movement and achieve a higher tensile strength than the 1Ni-AlSi10Mg sample. It is noteworthy that the pure AlSi10Mg sample showed a denser Si network than the 1Ni- and 3Ni-AlSi10Mg samples. However, the addition of Ni facilitated the precipitation of Si and promoted the continuity of the Si networks; thus, the continuity of the Si network was better in the 1Ni- and 3Ni-AlSi10Mg samples, as shown in Figure 9, which might facilitate the pinning of dislocations at Si networks. Moreover, the Al_3_Ni nanoparticles were not only distributed at the Si network boundaries but were also distributed inside the cells. The transfer of the load to the Al_3_Ni nanoparticles was possible by the outstanding interfacial bonding between *α*-Al and Al_3_Ni. These inner Al_3_Ni nanoparticles were conducive to producing and storing dislocations inside cells; thus, the tensile strength was raised by the strain fields generated by deformation-induced dislocations. In addition, dislocations within the cells cannot transmit across the dislocation walls inside the Si network. Thus, they accumulate within the cells, resulting in a high strain-hardening rate. During the tensile testing, a high strain-hardening rate helped to prevent the necking of the material, thus improving the ductility of the samples. This is also the main reason why the 1Ni- and 3Ni-AlSi10Mg samples exhibit better elongation than the pure AlSi10Mg sample.

However, for the 5Ni-AlSi10Mg sample, an inferior tensile strength of 340.72 MPa and elongation of 4.22% were obtained. The poor mechanical properties should be due to its low relative density of 92.47 ± 0.88%, resulting from the severe balling and over-burning. In addition, the ultrafine Si network in the 5Ni-AlSi10Mg sample might also prevent the plastic deformation of the Al matrix when stretching. For the 5Ni-AlSi10Mg sample, the Al cells cannot allow stable void growth since they are subjected to the high stress transferred from the Si network [32].

Figure 12b shows the Vickers-hardness values of Ni/AlSi10Mg samples with different Ni contents. All of the Ni/AlSi10Mg samples showed higher Vickers-hardness values than the pure AlSi10Mg sample. The 3Ni-AlSi10Mg sample showed the highest Vickers-hardness value among all the samples. The increased hardness value of the Ni/AlSi10Mg samples should be reasonably attributed to the formation of Al_3_Ni nanoparticles and the precipitation of Si from supersaturated Al matrix toward pre-existing Si networks, since the Al_3_Ni nanoparticles and Si phases have approximately 700 HV and 1000 HV Vickers hardness, respectively. The slightly lower Vickers-hardness value of the 5Ni-AlSi10Mg sample than the 3Ni-AlSi10Mg sample should be related to its comparably low relative density.

**Figure 14:**
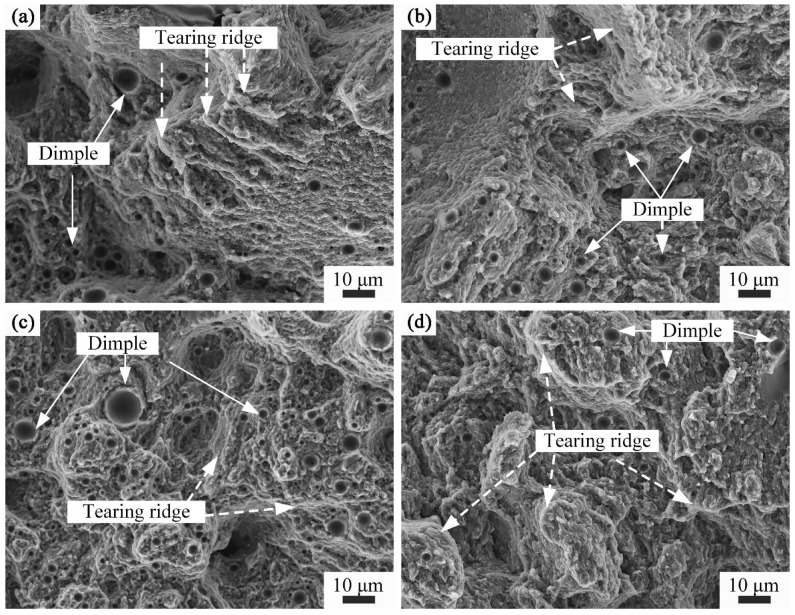
Fracture morphologies of Ni/AlSi10Mg samples with different Ni contents: (**a**) 0 wt.%, (**b**) 1 wt.%, (**c**) 3 wt.%, and (**d**) 5 wt.%.

Figure 14 shows the tensile fracture morphology of Ni/AlSi10Mg samples with different Ni contents. The fracture almost occurred along with the cleaved and fractured Si particles at the Si network boundaries, which could work as a stress-concentration site to induce crack initiation and cause the tear to spread along the *α*-Al cells. It is also evident that both the average size and number of dimples increased with increasing the Ni contents. All the Ni/AlSi10Mg samples show quasi-cleavage fracture features since tear ridges, cleavage steps, and small dimples can be clearly found in their fracture morphologies. Quasi-cleavage should not think to be a single event of a semi-brittle fracture process. Actually, it involves frequent microcrack nucleation and propagation events. Namely, the quasi-cleavage is a mixture of both ductile and brittle characteristics. The dimples were created after the micro-voids produced, grew, and subsequently coalesced with each other, as this benefited the plastic deformation of the samples. As shown in Figure 14c, abundant coarse dimples were found on the fracture surface of the 3Ni-AlSi10Mg sample, indicating the high ductility of the sample.

However, for the 5Ni-AlSi10Mg sample, the size and amount of the dimples decreased obviously. The fractography of the 5Ni-AlSi10Mg sample exhibited a brittle cleavage fracture with many tear ridges, and this was responsible for its low ductility. The brittleness should be due to the excessive damage nucleated on the Si network since the Si network was mostly interconnected in the 5Ni-AlSi10Mg sample and cannot bear high strain before failure.

### 3.4. Friction Coefficient and Wear Property

**Figure 15:**
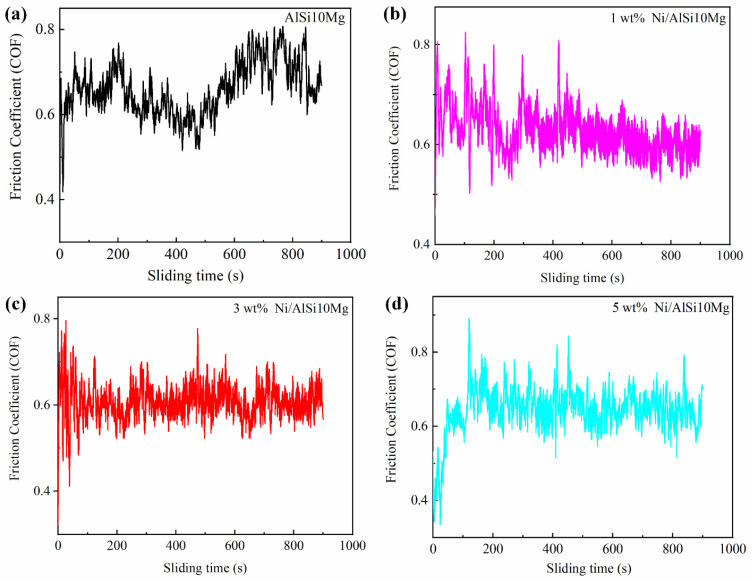
The curves of the friction coefficient versus sliding time of Ni/AlSi10Mg samples with different Ni contents.

Figure 15 shows the variation of friction coefficient versus sliding time for Ni/AlSi10Mg samples with different Ni contents. The average friction coefficients were 0.66, 0.628, 0.608, and 0.646, respectively, for the pure AlSi10Mg, 1Ni-AlSi10Mg, 3Ni-AlSi10Mg, and 5Ni-AlSi10Mg SLMed samples. The measured friction coefficients were somewhat variable during the sliding process. As shown in Figure 15a, the friction-coefficient curve of the AlSi10Mg test without Ni fluctuates greatly. Since the Si network in the pure AlSi10Mg sample was relatively discontinuous, cracks and delamination were likely to occur under the action of the external load, leading to a higher wear rate and fluctuation of the friction coefficient. As the Ni contents increase, the fluctuation of the friction coefficient of the composite sample gradually stabilizes and shows a horizontal line. Especially when the Ni content is 3 wt.%, the peak value of the friction coefficient curve is small and the number is small, and the curve is more stable as a whole, as shown in Figure 15c. The stable friction coefficient was due to the uniformly dispersed Al_3_Ni nanoparticles and interconnected Si network in the 3Ni-AlSi10Mg sample, leading to high strength and strong deformation resistance.

**Figure 16:**
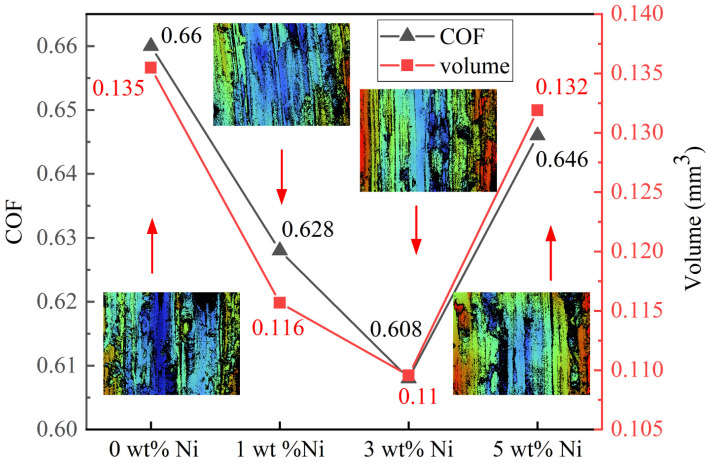
The average friction coefficient and wear volume of Ni/AlSi10Mg samples with different Ni contents. The inserts are the white-light interferogram of the worn surfaces.

**Figure 17:**
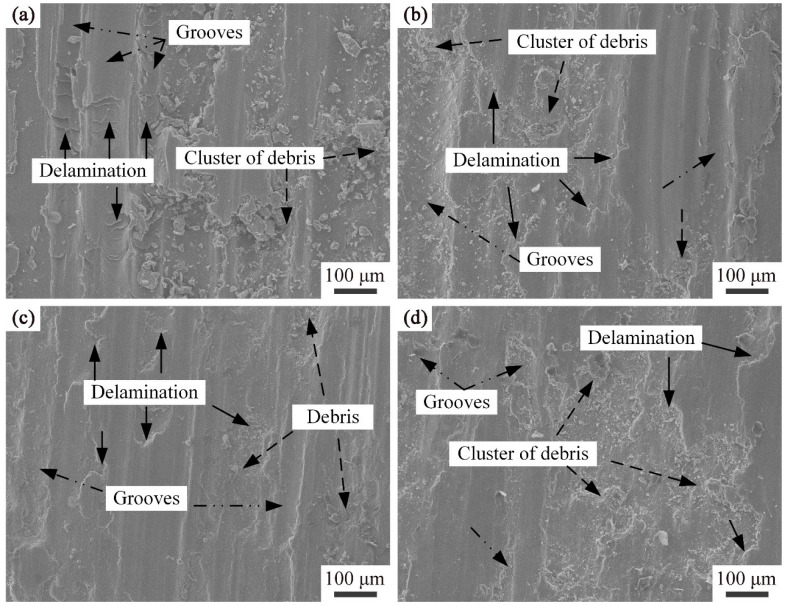
Worn surfaces of Ni/AlSi10Mg samples with different Ni contents: (**a**) 0 wt.%, (**b**) 1 wt.%, (**c**) 3 wt.%, and (**d**) 5 wt.%.

Figure 16 shows the relationship between the average friction coefficient and actual wear volumes of Ni/AlSi10Mg samples with different Ni contents. Figure 17 shows the worn surfaces of the samples. The friction coefficient and wear volume are basically positively correlated. Both the friction coefficient and the wear volumes decrease with Ni contents up to 3 wt.% and increase thereafter. The minimum values of the friction coefficient and wear volumes in the 3Ni-AlSi10Mg sample were 0.608 and 0.11 mm^3^, which were 7.88% and 18.5% lower than those of the pure AlSi10Mg sample, respectively. The white-light interferogram morphologies of the abrasion of Ni/AlSi10Mg samples with different Ni contents are shown in inserts in Figure 16. The color transformation clearly shows the wear state of the samples with different Ni contents. Among them, the pure AlSi10Mg and 5Ni-AlSi10Mg samples exhibited the deepest sunken region with dark blue, which indicated the largest wear.

As shown in Figure 17, adhesion layers were visible in the worn tracks for all the investigated samples, indicating that adhesion wear was the primary mechanism. For the pure AlSi10Mg sample, the presence of irregularly shaped debris revealed the local severe deformation and delamination of the worn surface that occurred. The wear trajectory of the 1Ni-AlSi10Mg sample was relatively shallow, which corresponds to the low wear rate measured in Figure 16. The worn surface of the 3Ni-AlSi10Mg sample was smoother, with fewer debris and shallow grooves. This should be due to the higher relative density, as well as the Al_3_Ni nanoparticles and eutectic Si networks with strong resistance to local shear stress. However, some clusters of debris were observed in the 5Ni-AlSi10Mg sample, indicating that the resistance to local shear stress was decreased. Here we can conclude that the friction coefficient of the 5Ni-AlSi10Mg sample first stabilized at a small value and then rapidly increased due to the scratching of the wear scar surface by the debris. The wear-resistance ability was usually related to the hardness and relative density of the samples. The 5Ni-AlSi10Mg sample had a low relative density of 92.47 ± 0.88% due to severe balling and over-burning. The relatively poor densification behavior and Vickers hardness led to the worse worn surface condition of the 5Ni-AlSi10Mg sample, with considerable flake delamination.

## 4. Conclusions

In this study, Ni/AlSi10Mg samples containing 0%, 1%, 3%, and 5% Ni by weight were fabricated by using the SLM process. The effects of the Ni contents on the microstructure, phase identification, and mechanical properties of the samples were systematically investigated. The main conclusions are summarized as follows:

(1) With the same SLM process parameters, the relative densities of the Ni/AlSi10Mg samples gradually decreased from 99.07% to 92.47% as the Ni contents increased. The energy absorption of the composites was improved with the increasing Ni contents, resulting in the balling and over-burning defects. The balling and over-burning defects led to a low relative density, especially for the 5Ni-AlSi10Mg sample.
(2) The addition of Ni contents in AlSi10Mg samples can refine the size of the Si networks and can also be beneficial to the precipitation of Si, which facilitates the continuity of the Si networks. The in situ reaction of the Ni and aluminum matrix produced Al_3_Ni nanoparticles in the SLM process. HRTEM verified that the interface between the Al_3_Ni nanoparticle and the Al matrix was well-bound.
(3) The relative density of the SLMed Ni/AlSi10Mg decreased when increasing the Ni contents. However, the mechanical performances of the Ni/AlSi10Mg samples were not solely determined by their relative density. The addition of Ni nanoparticles facilitated the precipitation of Si and promoted the continuity of the Si networks; thus, the continuity of the Si network was better in the 1Ni- and 3Ni-AlSi10Mg samples, as compared to the pure AlSi10Mg samples. The precipitated Si particles facilitated the pinning of dislocations at Si networks. Moreover, the Al_3_Ni nanoparticles were able to store dislocations inside *α*-Al cells. Thus, the 1Ni- and 3Ni-AlSi10Mg samples showed better tensile properties than the pure AlSi10Mg sample. Moreover, due to the higher Vickers hardness of the Al_3_Ni nanoparticles and Si phases, the Vickers-hardness values of the 1Ni- and 3Ni-AlSi10Mg samples were also higher than those of the pure AlSi10Mg sample. In addition, dislocations within the cells cannot transmit across the dislocation walls inside the Si network. Thus, they accumulate within the cells, resulting in a high strain-hardening rate. During the tensile testing, the high strain-hardening rate helped to prevent the necking of the material, thus improving the ductility of the samples. This is also the main reason why the 1Ni- and 3Ni-AlSi10Mg samples exhibit better elongation than the pure AlSi10Mg sample. As for the 5Ni-AlSi10Mg samples, serious balling and over-burning could be observed both in the top and lateral surfaces of the samples due to the addition of Ni nanoparticles which decreased the pavement quality layer by layer. As a result, the tensile strength and wear properties of the 5Ni-AlSi10Mg samples were inferior to the 1Ni- and 3Ni-AlSi10Mg samples. As the stress was concentrated in the Si networks, the most interconnected Si networks in the 5Ni-AlSi10Mg sample cannot bear high strain before failure due to the excessive damage nucleated on the Si networks, leading to the degraded elongation of the samples. To sum up, only the suitable Ni contents were beneficial to obtaining a high printing quality with relatively high density and high mechanical properties of the AlSi10Mg samples.
(4) The 3Ni-AlSi10Mg samples exhibited excellent overall performances, including a tensile strength of 401.15 ± 7.97 MPa, elongation of 6.23 ± 0.252%, Vickers hardness of 144.06 ± 0.81 HV, friction coefficient of 0.608, and wear volumes of 0.11 mm^3^, thus outperforming the pure AlSi10Mg samples (372.05 ± 1.64 MPa, 5.84 ± 0.269%, 123.22 ± 1.18 HV, 0.66, and 0.135 mm^3^).

The results revealed that the typical structure of the eutectic Si network of SLM-produced AlSi10Mg alloys can be modified by adjusting the contents of Ni in the Ni/AlSi10Mg composites. The improved mechanical properties of the 3Ni-AlSi10Mg samples indicated that Ni could be a potential addition in improving the mechanical properties of the AlSi10Mg alloys by SLM.

## Figures and Tables

**Table 1:** Printing parameters of Ni/AlSi10Mg samples with different Ni contents.

Laser Power	Scanning Speed	Hatch Spacing	Layer Thickness
350 W	1850 mm/s	130 μm	30 μm

**Table 2:** Mechanical properties of Ni/AlSi10Mg samples with different Ni contents.

Ni Content	Tensile Strength (MPa)	Elongation (%)	Relative Density (%)	Vickers Hardness (HV)
0 wt.% Ni	372.05 ± 1.64	5.84 ± 0.269	99.07 ± 0.46	123.22 ± 1.18
1 wt.% Ni	377.33 ± 4.02	5.90 ± 0.265	97.29 ± 0.84	136.3 ± 1.63
3 wt.% Ni	401.15 ± 7.97	6.23 ± 0.252	96.56 ± 0.74	144.06 ± 0.81
5 wt.% Ni	340.72 ± 8.12	4.22 ± 0.247	92.47 ± 0.88	140.08 ± 0.87

## Data Availability

The data that support the findings in this paper are available from the corresponding authors upon reasonable request.
